# Transcriptome analysis reveals early activation of MAPK pathways involved in the resistance of *Setaria italica* against *Pyricularia setariae*

**DOI:** 10.3389/fpls.2025.1676191

**Published:** 2025-11-27

**Authors:** Shang Feng, Jia Liu, Xinpeng Han, Tingting Liu, Saiya Wang, Shaoshuai Xin, Zhimin Hao, Zhiyong Li

**Affiliations:** 1 Key Laboratory of Genetic Improvement and Utilization for Featured Coarse Cereals (Co-construction by Ministry and Province), Institute of Millet Crops, Hebei Academy of Agriculture and Forestry Sciences Shijiazhuang, Hebei, China; 2 Key Laboratory of Minor Cereal Crops of Hebei Province, Ministry of Agriculture and Rural Affairs, Shijiazhuang, Hebei, China; 3 State Key Laboratory of North China Crop Improvement and Regulation, Hebei Agricultural University, Baoding, Hebei, China

**Keywords:** foxtail millet blast, *Pyricularia setariae*, transcriptome analysis, resistant mechanism, pathogen-host interaction

## Abstract

Millet (Setaria italica) is an important food crop in China, and its yield and quality are often severely threatened by foxtail millet blast (the pathogen is *Pyricularia setariae*). Currently, the molecular mechanism of millet’s resistance to *P. setariae*, especially the key regulatory links of the early signal transduction pathway, is still poorly understood. The mitogen-activated protein kinase (MAPK) signaling pathway plays a conserved and crucial role in plant immunity, rapidly activating downstream defense genes to respond to pathogen invasion. However, the specific function of this pathway in the interaction between S. italica and *P. setariae* and its contribution to disease resistance remain unclear. To clarify the role of the MAPK pathway in millet’s disease resistance process, this study selected the high-resistance variety ZGB and the high-susceptibility variety JG19, and used RNA-seq technology to systematically analyze the dynamic changes in the leaf transcriptome after inoculation with *P. setariae*. KEGG pathway analysis showed that in the resistant variety, genes related to photosynthesis and ribosomes were downregulated in the early stage of infection and then rapidly upregulated, demonstrating a strong self-repairing ability; while in the susceptible variety, these genes were continuously downregulated, resulting in severe damage to physiological functions. Further studies have shown that this regulatory process may be closely related to the MAPK signaling pathway. This study focuses on expression differences of MAPK pathways and their downstream transcription factors in resistant and susceptible varieties aiming to reveal regulatory roles of this pathway in early immune responses of millet while providing theoretical basis as well as genetic resources for analyzing mechanisms underlying disease resistance.

## 1 Introduction

Foxtail millet (*Setaria italica*), which originated in China, represents a pivotal crop in agricultural history. It is not only the first multi-grain crop to undergo complete genome sequencing but also one of the earliest domesticated crops in the region (Lydia Pramitha et al., 2022). Its compact genome and rapid growth cycle render it an ideal monocotyledonous model organism for genetic and physiological studies. As a C4 plant, it provides unique insights into mechanisms of efficient photosynthetic energy conversion and stress tolerance (Panchal et al., 2023). Foxtail millet exhibits a balanced nutritional profile, being rich in protein, fat, and vitamins, making it valuable for both human consumption and animal feed. Furthermore, its environmental sustainability, high yield potential, and drought resilience position it as a promising candidate for future biofuel production. In the context of escalating environmental challenges, its role as a strategic reserve crop holds substantial societal significance. Foxtail millet’s short growth duration, minimal water requirements, and robust adaptability to adverse climatic conditions enable it to outperform major cereals such as rice and maize on marginal lands (Kumar and Singh, 2024). Its versatility allows it to thrive under diverse agricultural climates, particularly in semi-arid regions of Africa and Asia, where it can contribute to mitigating the impacts of extreme weather events (Nagaraja et al., 2024). However, in China’s primary grain-producing areas, foxtail millet faces a severe threat from the disease known as foxtail millet blast.

The causal agent of millet blast is *Pyricularia setariae*. Its conidiophores emerge singly or in clusters on the lesion surface, are colorless, and typically unbranched. The conidia are pyriform (pear-shaped), colorless, and usually possess two internal septa (Hasan et al., 2024; Longya et al., 2020; Persaud et al., 2007). The optimal temperature range for pathogen growth is 25-28°C, with survival possible at extreme temperatures up to 38°C. At 15°C, growth significantly slows, yet spore germination remains viable under these conditions. High humidity is essential for spore germination, and disease severity tends to increase in high-temperature and high-humidity environments. Upon landing on millet leaves or other plant parts, spores germinate in the presence of moisture, producing 1–2 germ tubes. These germ tubes form appressoria upon contact with the host surface, followed by the development of invasive hyphae. Hyphae can penetrate epidermal cells directly or enter leaf tissues through stomata. In panicles, the pathogen frequently invades via branches of spikelet pedicels, while at stem nodes, invasion predominantly occurs through outer leaf sheaths. The pathogen’s reproduction cycle within leaves lasts approximately 7–10 days, which can be shortened to 5–6 days under favorable conditions. As millet tissues progressively wilt, conspicuous lesions develop. Simultaneously, the pathogen releases new conidiophores through stomata and produces additional spores, initiating a reinfection cycle (Zhao et al., 2014). This process results in a yield reduction of 20%-30%, with severe cases leading to losses of 50%-70% (Li et al., 2016). Compounded by the absence of durable resistant varieties and the widespread practice of continuous cropping, millet blast has emerged as a major threat to millet production in China. Environmental pollution, chemical residue, and the emergence of drug-resistant strains further underscore the need for innovative control strategies (Sharma et al., 2014; Tian et al., 2021). Presently, research on millet blast primarily focuses on the biological characteristics of the pathogen. Although significant progress has been made in understanding the host resistance mechanisms in millet, substantial knowledge gaps remain, particularly in the characterization of resistance mechanisms among millet germplasm exhibiting divergent resistance and susceptibility traits in response to *P. setariae*, a domain that is still poorly understood. This research gap may stem from the scarcity of resistant varieties, thereby constraining deeper exploration of the interaction mechanisms between millet and the pathogen.

This study utilized the disease-resistant variety ZGB and the disease-susceptible variety JG19 as research materials. Through transcriptome sequencing technology, leaf samples from both varieties were analyzed at multiple time points post-inoculation with the pathogen *P. setariae* to comprehensively investigate dynamic changes in gene transcription. The study delved into metabolic pathways and genes closely associated with millet’s disease resistance mechanisms. It was observed that the disease-resistant variety ZGB significantly upregulated the expression of photosynthesis- and ribosome-related genes following pathogen infection, whereas the disease-susceptible variety JG19 exhibited no such upregulation. Additionally, during the mid-stage of infection, transcription factors (e.g., WRKY, ERF, and MYB) and genes involved in the MAPK signaling pathway were markedly enriched in ZGB. These findings indicate that ZGB effectively combats pathogen infection by preemptively activating its repair mechanisms and disease resistance-associated gene expression. This research not only provides valuable insights for a deeper understanding of the molecular interactions between millet and *P. setariae* but also establishes a robust theoretical foundation for millet disease resistance breeding, with potential implications for the development and application of disease-resistant millet varieties (Panchal et al., 2023).

## 2 Materials and methods

### 2.1 Strains, plants and culture conditions

The *P. setariae -3* (*Ps3*) strain utilized in this research has been deposited at the Institute of Millet Crops, Hebei Academy of Agriculture and Forestry Sciences. The disease-resistant planting resource ZGB of *S. italica* originates from Guangling grain resources in Shanxi Province. The susceptible variety JG19 of *S. italica* is maintained at the Institute of Millet Crops, Hebei Academy of Agriculture and Forestry Sciences. Fungi are more likely to produce spores on oat tomato agar (OTA: tomato sauce 150 mL/L, raw oatmeal 40 g/L, CaCO_3_0.6 g/L, agar 18.75 g/L) when cultivated at 26°C under light conditions. The ZGB and JG19 were cultured in a growth at chamber at 28°C under a photoperiod of 14 h light and 10 h dark with a light intensity of 6000 lux. (28°C is its optimal growth temperature) (Li et al., 2015).

### 2.2 Sample collection

When the *S. italica* seedlings reached the 6–7 leaf stage(three weeks), suspensions at a concentration of 10^5^ spores/mL were evenly sprayed on the foxtail millet leaves. The plants were kept moist under dark conditions for 24 hours (humidified with a humidifier) and then placed in an incubator at 28°C for further cultivation. Combined with the determination of the disease index, where disease index = 100×Σ(levels of diseased leaves×representative values)/(total number of surveyed leaves×highest-level representative value), determine the resistance and susceptibility traits of millet varieties. Leaf samples from *S. italica* were collected at 0 h, 12 h, 24 h, 36 h, 48 h, and 72 h after inoculation, with one leaf collected from each basin. A total of eight leaves constituted one replicate. Three biological replicates were established. The collected leaves were quickly placed into liquid nitrogen and then stored at -80°C. These samples were utilized for subsequent transcriptome sequencing and qRT-PCR analysis (Liu et al., 2024).

### 2.3 RNA extraction

Total RNA was extracted from S. italica using the E.Z.N.A.^®^ Fungal RNA Kit (Omega Bio-Tek). The concentration and purity of the extracted RNA were detected using Nanodrop2000. The integrity of the RNA was examined by agarose gel electrophoresis, and the RQN value was determined by Agilent5300. For a single library construction, the total amount of RNA should be 1 μg, with a concentration of ≥ 30 ng/μL, RQN > 6.5, and the OD260/280 ratio ranging from 1.8 to 2.2. RNA purification, reverse transcription, library preparation, and sequencing were performed by Shanghai Majorbio Bio-pharm Biotechnology Co., Ltd.

### 2.4 Library preparation and RNA-seq

A strand-specific transcriptome library was constructed from 1 µg of total RNA using the Illumina^®^ Stranded mRNA Prep, Ligation kit. Key steps included: mRNA isolation via poly(A) selection with oligo(dT) beads, fragmentation, double-stranded cDNA synthesis using random hexamer primers, end repair/A-tailing/adapter ligation, size selection for 300–400 bp fragments with magnetic beads, and PCR amplification (10–15 cycles). Quantified libraries (Qubit 4.0) were sequenced on the NovaSeq X Plus platform (PE150) using the NovaSeq Reagent Kit. The original RNA-Seq data has been publicly available on NCBI BioProject, with the accession number SUB14926886.

### 2.5 RNAseq analysis and gene expression analysis

The raw paired-end reads were trimmed and quality controlled by Fastp (Chen et al., 2018)with default parameters. Then clean reads were separately aligned to the reference genome in orientation mode using HISAT2 (Kim et al., 2015). The mapped reads of each sample were assembled by StringTie (Pertea et al., 2015) in a reference-based approach. The original sequencing reads were filtered to remove adapters and low-quality sequences. Then, the high-quality paired-end reads were compared with the barley reference genome (Setaria italica Yugu1_T2T). The gene expression levels were quantified using the FPKM (Fragments Per Kilobase per Million mapped reads) value, and the calculation tool was Cufflinks. The reference genome information is as follows: the species is Setaria italica, the version is Yugu1_T2T, and the source website is http://111.203.21.71:8000/download.html. The gene differential expression analysis was based on the log2(FPKM) ratio, and all analyses included three biological replicates (Bolger et al., 2014). KEGG pathway enrichment analysis was conducted using the Python scipy package, and calculations were performed using Fisher’s exact test. To control for false positive rates in these calculations, BH (FDR) method was used for multiple testing corrections; corrected p-values (Corrected P-Value) were set at 0.05 as a threshold, defining KEGG pathways that met this condition as significantly enriched pathways among differentially expressed genes.

### 2.6 Overall data annotation and differentially expressed genes

A total of 36 samples were sequenced for transcriptome analysis, resulting in a total of 244.56 Gb of clean data. The amount of clean data for each sample was no less than 5.46 Gb, and the Q30 base percentage was higher than 96.49%. The reference genome information is as follows: the species is Setaria italica, the version is Yugu1_T2. The alignment efficiency of clean reads for each sample with the reference genome ranged from 95.57% to 97.97%. After obtaining the read counts of the genes, an analysis of gene expression differences between samples (≥2) was conducted to identify differentially expressed genes among samples, and then the functions of these differentially expressed genes were studied. The software used for differential expression analysis was DESeq2 (Love et al., 2014), and the default filtering criteria for significantly differentially expressed genes were: FDR < 0.05 & |log2FC| ≥ 1. When a gene met both conditions, it was regarded as a differentially expressed gene (DEG). Any gene with a standardized expression level (TPM) lower than 1 is considered a low-expression gene. Genes below this threshold are regarded as unreliable in the differential expression analysis and are filtered out before statistical tests are conducted. This conventional practice helps to reduce background noise and improve the reliability of the identified differentially expressed genes (DEGs). Software used: DESeq2 (http://bioconductor.org/packages/stats/bioc/DESeq2/).

### 2.7 RT-qPCR analysis

Gene expression levels were quantified by qPCR analysis using the 2×Universal SYBR Green Fast qPCR Mix (ABclonal) on the CFX96 Real-Time System (Bio-Rad), as previously described (Meng et al., 2022). The expression levels of the target genes(table1) were estimated using the 2*^−^*^ΔΔCT^ method, with the β-actin gene used as the internal control (Bustin et al., 2009; Zeng et al., 2020). The specific primers used in this study are listed in **Table S1**.

## 3 Results

### 3.1 Pathogenicity detection and transcriptome data validation of different varieties of foxtail millet

**Figure 1:**
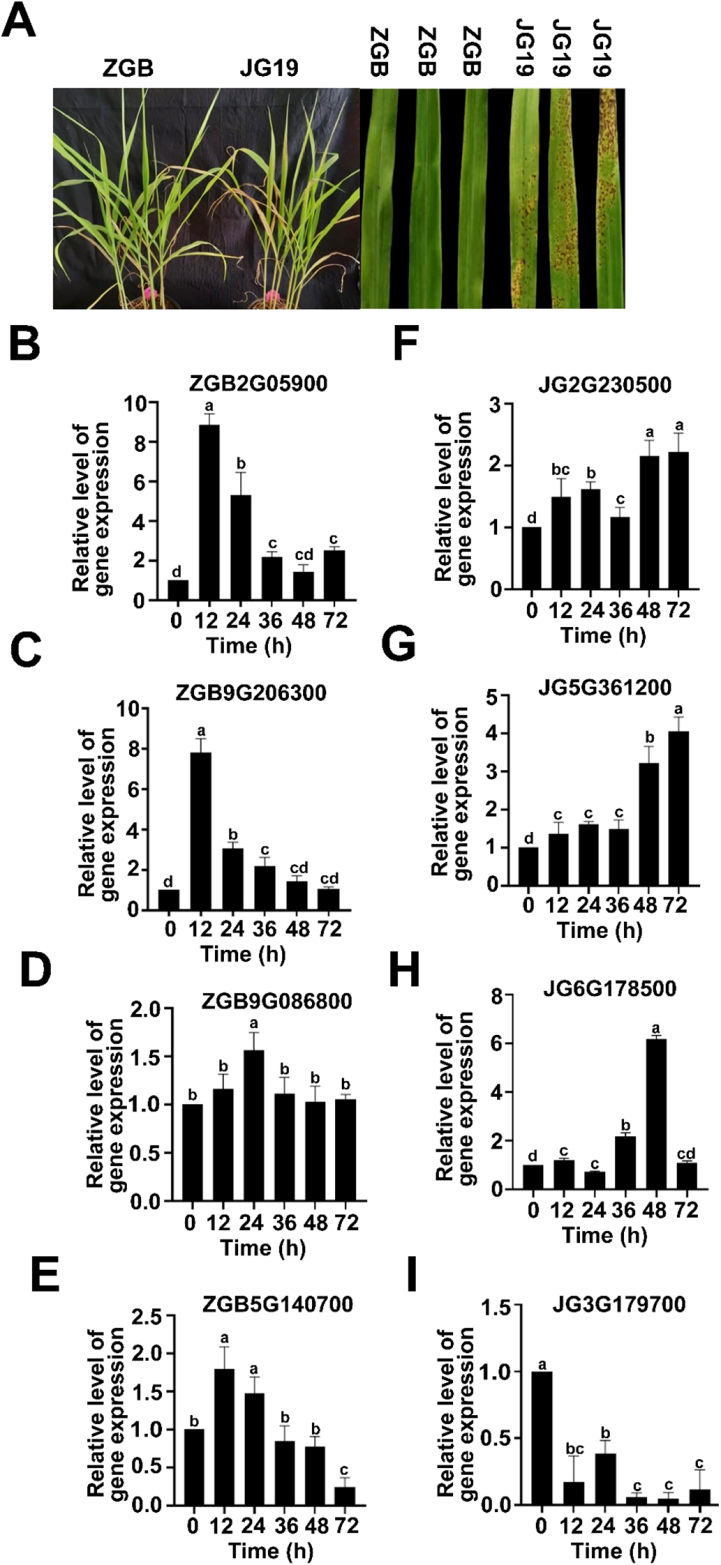
Pathogenicity detection and transcriptome data validation of different varieties of foxtail millet. Phenotypes of different varieties infected with *Ps3***(A)**. Real-time qPCR validation of representative genes at three time points in ZGB and JG19 during infection by *Ps3*. The left Y axis corresponds to the gene expression level in transcriptome sequencing shown in broken lines. The right Y axis corresponds to the relative gene expression level in qRT-PCR shown in a column. Reference gene: β-actin; n = 3**(B-I)**.

Millet blast, a fungal disease caused by *Pyricularia setariae* (*Ps3*), can occur at all growth stages of millet, with spike infections being particularly conspicuous at later phases and potentially leading to severe yield loss. In this study, we compared the resistant variety “Ziganbai (ZGB)” and the susceptible variety “Jigu 19 (JG19)” to investigate their contrasting responses to *Ps3* inoculation. As shown in **Figure 1A**, susceptible plants (JG19) developed extensive blast lesions on grain leaves, whereas ZGB plants exhibited only sporadic, non-expanding necrotic spots, indicating effective resistance. This phenotypic difference was quantitatively supported by the disease index—11.11 for ZGB versus 90.17 for JG19—confirming a significant disparity in blast resistance between the two varieties. There were significant differences in resistance and susceptibility traits between the two varieties, with ZGB being more resistant to than JG19. To validate the RNA-Seq results, eight DEGs were randomly selected for qRT-PCR analysis. These genes include the gene ZGB2G059000 encoding hypothetical protein SETIT, gene ZGB9G206300 encoding dof zinc finger protein 4, gene ZGB9G086800 encoding hypothetical protein SETIT and unknown function of the gene ZGB5G140700, gene JG2G230500 encoding ethylene-responsive transcription factor ERF109, gene JG5G361200 encoding trans-cinnamate 4-monooxygenase, gene JG6G178500 encoding nitrate reductase, gene JG3G179700 encoding hypothetical protein SETIT. The qRT-PCR results were compared with the Illumina RNA-Seq sequencing results, as shown in (**Figures 1B-I**). Except for ZGB5G140700, the expression trends of the other genes in qRT-PCR were fully consistent with the RNA-Seq results, with a concordance rate of 90.91%. These results confirm the high fidelity and consistency of the transcriptome analysis in this experiment.

### 3.2 Transcriptome data analysis

#### 3.2.1 Differential gene expression analysis

**Figure 2:**
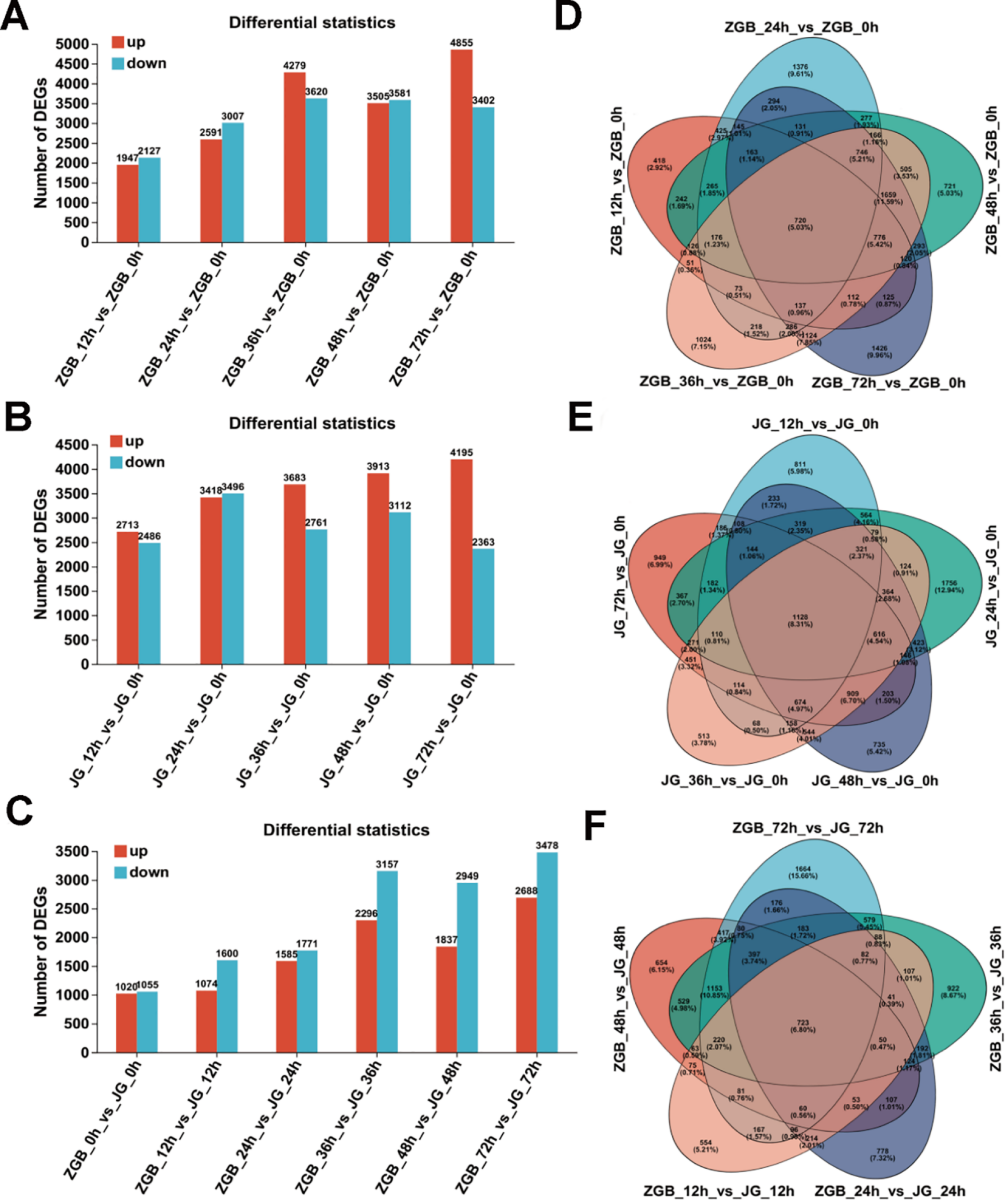
Differential Gene Expression and Venn Diagram Analysis. The significantly up-regulated and down-regulated differential genes in ZGB samples at various time points were compared to those at 0 h. The significantly up-regulated and down-regulated differential genes in JG samples at various time points were also compared to those at 0 h **(A, B)**. The significantly up-regulated and down-regulated differential genes were compared between ZGB and JG samples at the same time point **(C)**. Venn analysis of ZGB samples compared to 0 h samples at different time points; Venn analysis of JG samples compared to 0 h samples at different time points; Venn analysis of ZGB versus JG samples during the same period **(D-F)**.

The number of reads corresponding to each gene was determined by aligning to the reference genome, excluding low-expression genes. The results indicated that there were 1,947 up-regulated and 2,127 down-regulated genes in the ZGB samples at 12 h compared to 0 h. At 24 h, there were 2,591 up-regulated and 3,007 down-regulated genes, while at 36 h, these numbers increased to 4,279 and 3,620, respectively. Similarly, at 48 h, there were 3,505 up-regulated and 3,581 down-regulated genes, and at 72 h, 4,855 up-regulated and 3,402 down-regulated genes. Notably, during the 72-hour post-inoculation, the difference between up-regulated and down-regulated genes was the largest, totaling 1,453 genes (**Figure 2A**). Venn analysis identified 720 genes expressed consistently across all four time points: 418 genes were specifically expressed at 12 hip, 1,376 genes at 24 hip, 1,024 genes at 36 hip, 721 genes at 48 hip, and 1,426 genes at 72 hip (**Figure 2D**). The above results showed that the DEGs increased during the infection, indicating that the response accompanied by the infection time of the pathogen was more intense.

The differential gene expression of susceptible JG19 samples at various time points revealed the greatest disparity at the 72 hip stage, with 1,832 more up-regulated genes than down-regulated ones (**Figure 2B**). Similarly, for ZGB, the largest disparity in gene regulation occurred at 72 hip. Venn analysis of the up-regulated and down-regulated differential genes in JG19 across different time points identified 1,128 genes consistently expressed across these time points, a higher proportion than in ZGB. Specifically, 811 genes were expressed at 12 hip, 1,756 at 24 hip, 513 at 36 hip, and 735 at 48 hip in samples inoculated with *Ps3*. Additionally, 949 genes were specifically expressed at 72 hip in samples inoculated with *Ps3*. Compared to ZGB samples inoculated with *Ps3*, JG19 exhibited the highest number of specifically expressed genes at 24 hip, whereas ZGB showed the highest at 72 hip (**Figure 2E**). Initial analyses highlighted pronounced spatiotemporal differences in gene expression between susceptible JG19 and disease-resistant ZGB. These findings underscore considerable spatial and temporal variation in gene expression across infected varieties, suggesting these genes may be closely related to the plant’s response to pathogen infection.

#### 3.2.2 Venn and PCA analysis

By comparing and analyzing the differential gene expression of ZGB and JG19 among the tested stages, the highest number of up-regulated and down-regulated genes was observed at 72 hip (**Figure 2C**). Venn analysis identified 1,664 specifically expressed genes in the differential genes of ZGB_72h vs JG_72h, a value surpassing that of other periods, while only 723 genes were commonly expressed across different periods (**Figure 2F**). These results indicate that the response of grain leaves to *Ps3* infection represents a complex and dynamic process, with some genes participating throughout the response and others active only at specific stages. Additionally, some genes exhibit consistently strong responses, while others show minimal activity.

**Figure 3:**
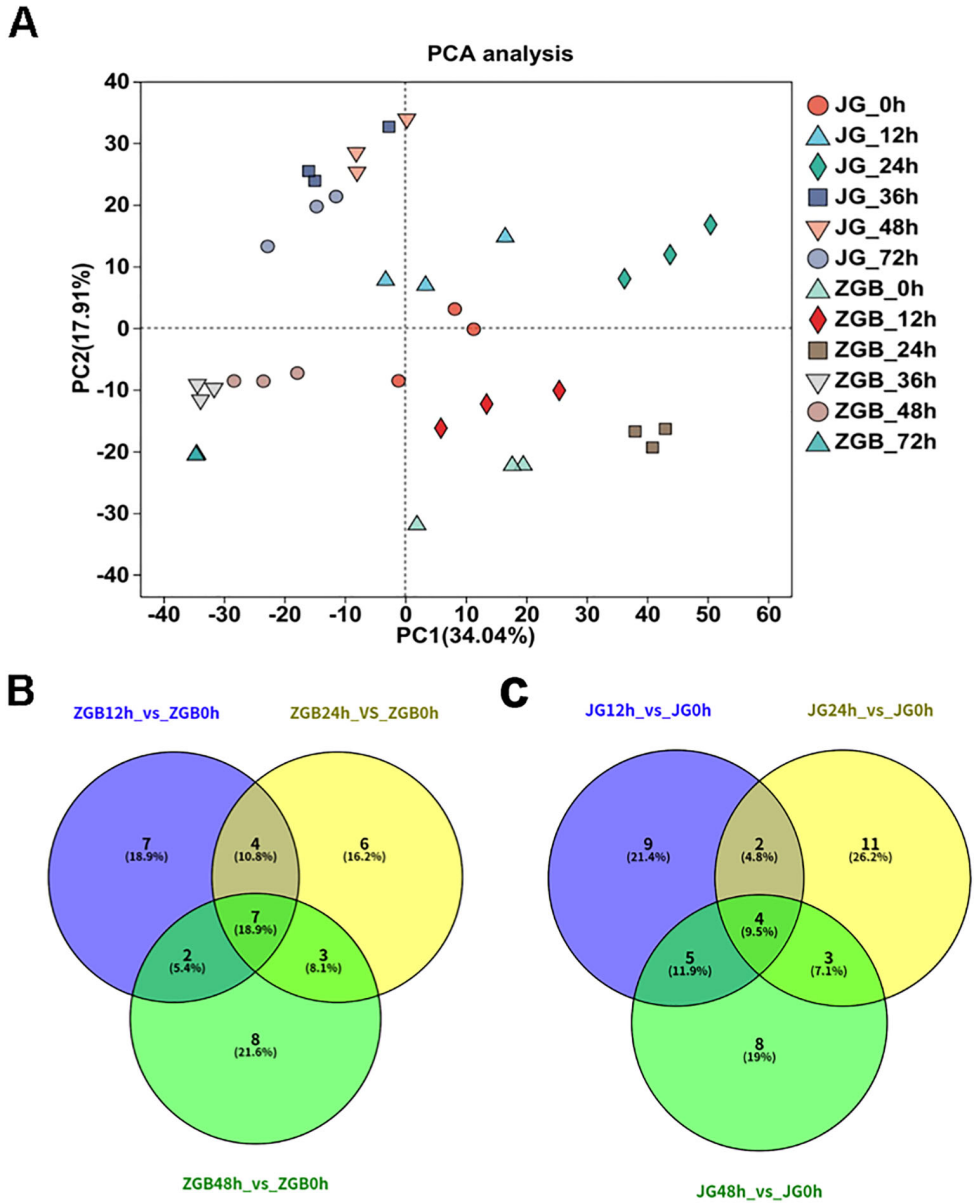
PCA and Venn analysis. PCA analysis of different varieties and periods **(A)**. Venn analysis of KEGG metabolic pathways in different varieties and stages**(B, C)**.

The results of PCA analyses of all samples showed (**Figure 3A**) that the results of the PCA revealed distinct clustering patterns among the samples. Replicate samples under the same treatment conditions were tightly grouped, indicating high reproducibility. However, the distribution of millet samples varied noticeably across different time points and between the two varieties. Specifically, samples from 0 h and 12 h post-inoculation formed dense clusters, as did those from 24 h. Similarly, samples from 36 h to 72 h also clustered together, yet the overall clustering tendencies of these three groups (0–12 h, 24 h, and 36–72 h) differed significantly. Moreover, a clear separation was observed between the two varieties. Based on these distinct temporal patterns, we defined 12 h, 24 h, and 48 h as the early, middle, and late stages, respectively, for subsequent analysis.

### 3.3 Analysis of KEGG enrichment of differential genes

In recent years, transcriptome sequencing (RNA-Seq) technology has been widely used in the study of molecular mechanisms during host plant-pathogen interactions to reveal changes in the expression of a series of relevant genes in the cell after pathogen infested the plant, which helps to explore the pathogenic mechanism of pathogens.

KEGG analyses were performed on the pre-, mid- and post-phase stages, and the 20 metabolic pathways with the highest enrichment were selected for Venn analysis. In ZGB, seven metabolic pathways were significantly enriched throughout the whole period(Cysteine and methionine metabolism, Pyruvate metabolism, Purine metabolism, Glyoxylate and dicarboxylate metabolism, Carbon fixation in photosynthetic organisms, Valine, leucine and isoleucine degradation, Butanoate metabolism), while seven were significantly enriched only in the pre-period(ribosome, glycerophospholipid metabolism, fructose and mannose metabolism, carotenoid biosynthesis, sphingolipid metabolism, ubiquinone and other terpenoid-quinone biosynthesis, inositol phosphate metabolism), six in the mid-period(photosynthesis, photosynthesis - antenna proteins, beta-Alanine metabolism, ribosome biogenesis in eukaryotes, arginine and proline metabolism, tyrosine metabolism), and eight in the late-period (sulfur metabolism, monobactam biosynthesis, valine, leucine and isoleucine biosynthesis, citrate cycle (TCA cycle), circadian rhythm – plant, arginine biosynthesis, glycerolipid metabolism, glycine, serine and threonine metabolism) (**Figure 3B**). In JG19, four metabolic pathways were significantly enriched throughout the entire period (Purine metabolism, pentose phosphate pathway, starch and sucrose metabolism, glyoxylate and dicarboxylate metabolism), nine metabolic pathways were significantly enriched only in the early period(Ribosome biogenesis in eukaryotes, sulfur metabolism, histidine metabolism, phosphatidylinositol signaling system, ascorbate and aldarate metabolism, thiamine metabolism, Vitamin B6 metabolism, folate biosynthesis, pantothenate and CoA biosynthesis), eleven were significantly enriched only in the middle period(Photosynthesis - antenna proteins, Photosynthesis, alpha-Linolenic acid metabolism, Carotenoid biosynthesis, Arginine and proline metabolism, Ribosome, Amino sugar and nucleotide sugar metabolism, Linoleic acid metabolism, Butanoate metabolism, Arginine biosynthesis, Lysine biosynthesis), and eight were significantly enriched only in the late period(Valine, leucine and isoleucine degradation, phenylalanine, tyrosine and tryptophan biosynthesis, terpenoid backbone biosynthesis, glycine, serine and threonine metabolism, fructose and mannose metabolism, citrate cycle (TCA cycle), ubiquinone and other terpenoid-quinone biosynthesis, galactose metabolism)(**Figure 3C**).Two metabolic pathways were significantly enriched throughout the entire period in both ZGB and JG19. They were ‘Purine metabolism’ and ‘Glyoxylate and dicarboxylate metabolism’, respectively. We therefore hypothesized that these two pathways played a role throughout the entire stage of infestation by the pathogen.

**Figure 4:**
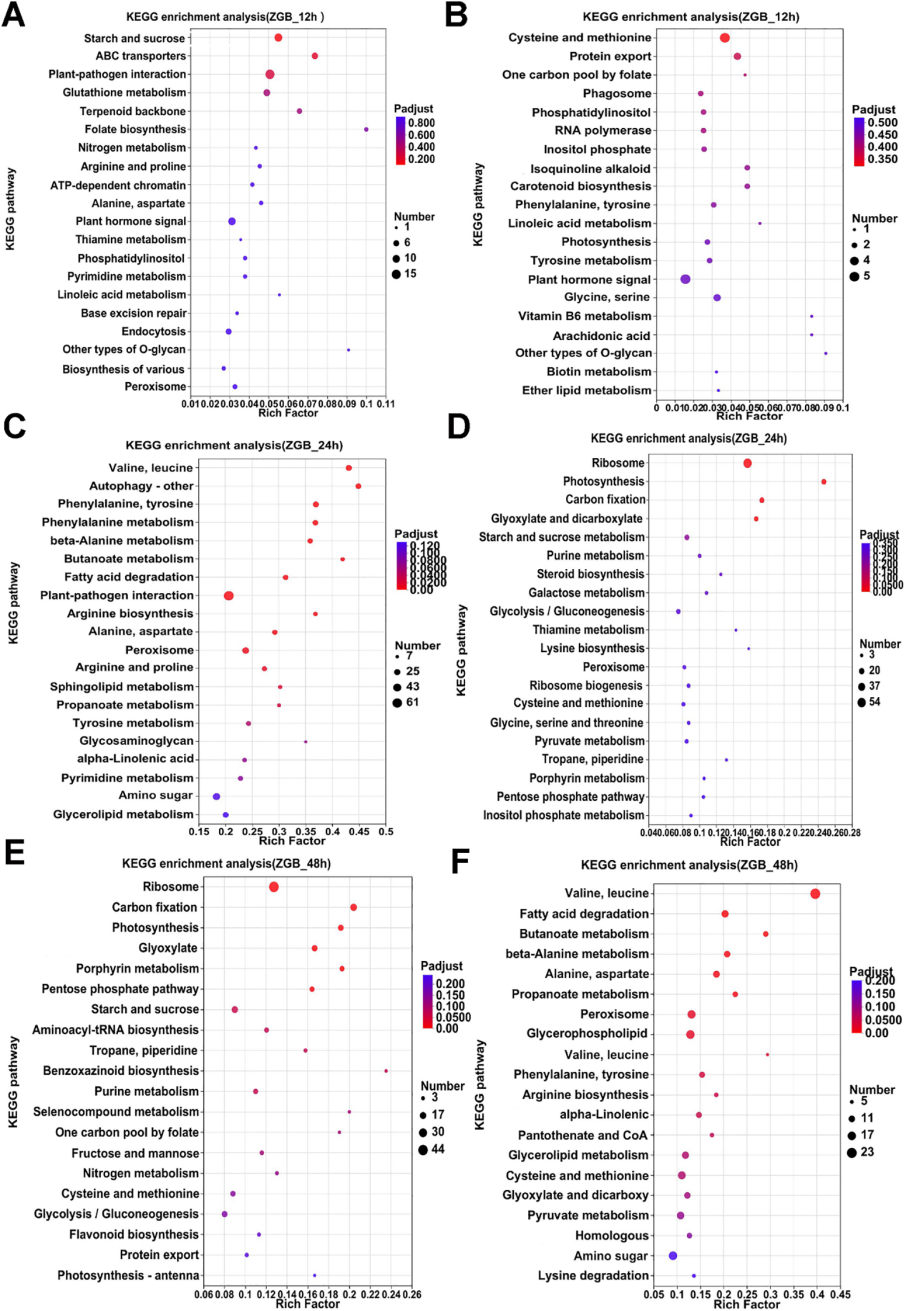
Functional enrichment analysis of differentially expressed KEGG genes in different varieties. KEGG functional enrichment analysis of ZGB DGEs **(A-F)**.

**Figure 5:**
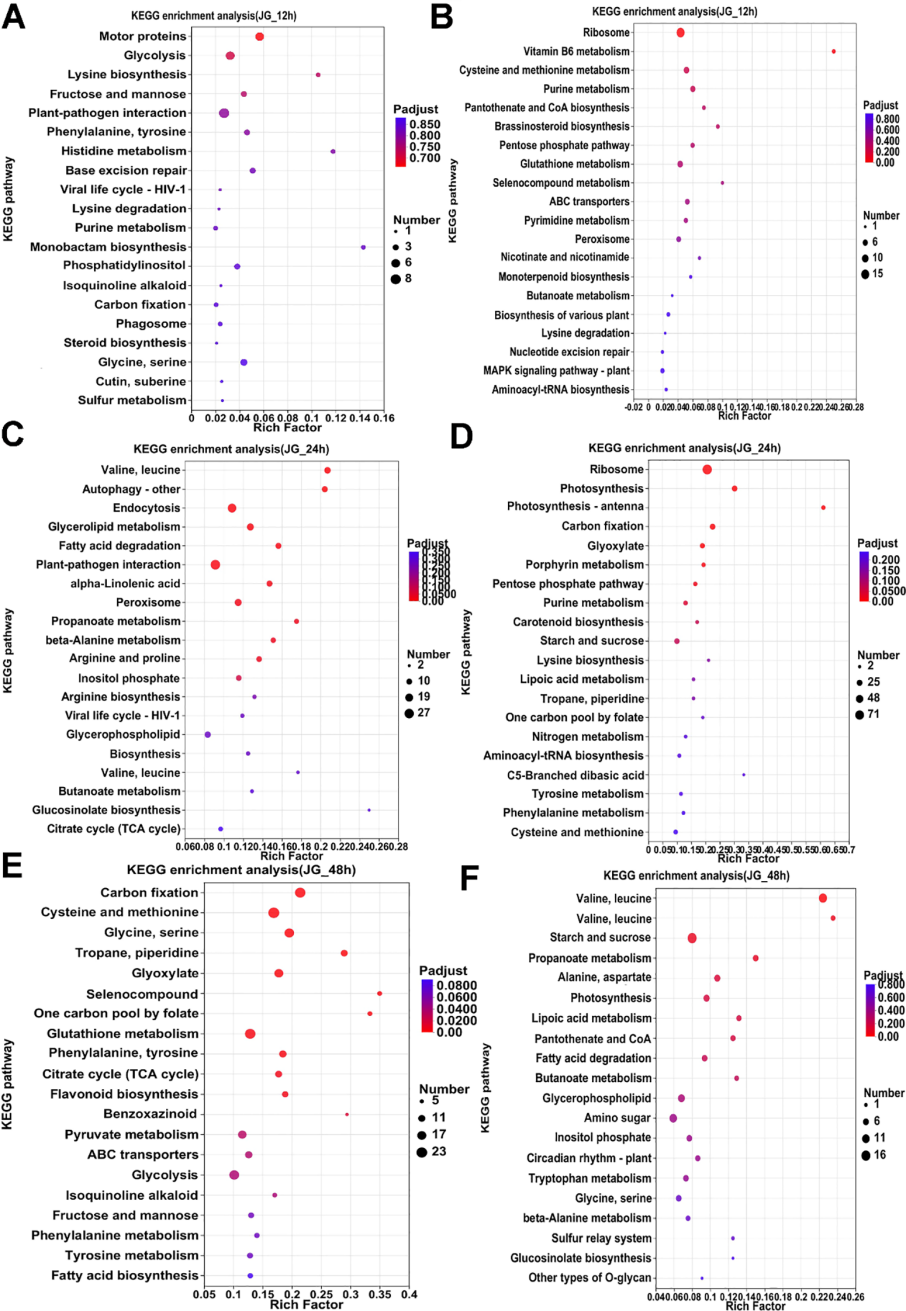
Functional enrichment analysis of differentially expressed KEGG genes in different varieties. KEGG functional enrichment analysis of JG19 DGEs **(A-F)**.

Analyzing the enrichment of differential genes in KEGG pathway during the pre-, mid- and post-phase stages of the two varieties (**Figures 4**, **5**), it can be seen that the differential genes of ZGB and JG19 differed greatly in the two pathways of ‘Ribosome’ and ‘Photosynthesis’, with genes related to these two pathways being significantly down-regulated in JG19 variety and significantly up-regulated in ZGB variety at a later stage. In addition, the genes related to ‘Glutathione metabolism’, ‘Phenylalanine metabolism’ and ‘Tyrosine metabolism’ were significantly down-regulated in the JG19 variety, whereas they were significantly up-regulated in the ZGB variety at a later stage(48hip).

Furthermore, genes related to pathways involving amino acid metabolism (such as “glutathione metabolism”, “phenylalanine metabolism”, and “tyrosine metabolism”) were significantly upregulated in the resistant variety ZGB at the early stage of infection, while they were significantly upregulated later in the susceptible variety JG19. In contrast, genes related to “starch and sucrose metabolism” were significantly downregulated in both ZGB and JG19 at the middle stage, but significantly upregulated in ZGB at the early stage and not significantly upregulated in JG19.”Purine metabolism” was significantly upregulated in ZGB in the middle and early stages, while it was downregulated in JG19 at the same period. “Nitrogen metabolism” was significantly upregulated in ZGB at the early stage and downregulated in the middle stage of JG19. “Alanine, aspartate, and glutamate metabolism” was significantly upregulated at the early stage and downregulated at the later stage in ZGB, while only significantly downregulated at the later stage in JG19.”Biological synthesis of various plant secondary metabolites” was significantly upregulated in ZGB at the early stage and downregulated at the early stage of JG19. “Peroxisome” was significantly upregulated at the early stage and downregulated at the later stage in ZGB, while it was significantly downregulated at the early stage and significantly upregulated at the middle stage in JG19. “ABC transporters” were significantly upregulated at the early stage in ZGB and downregulated at the middle stage and significantly upregulated at the later stage in JG19.”Plant-pathogen interaction” related genes were significantly upregulated in both ZGB and JG19 at the early and late stages, but only significantly upregulated at the early stage in JG19. “Glutathione metabolism” was significantly upregulated at the early stage in ZGB and significantly downregulated at the early stage of JG19, but significantly upregulated only at the later stage.

**Figure 6:**
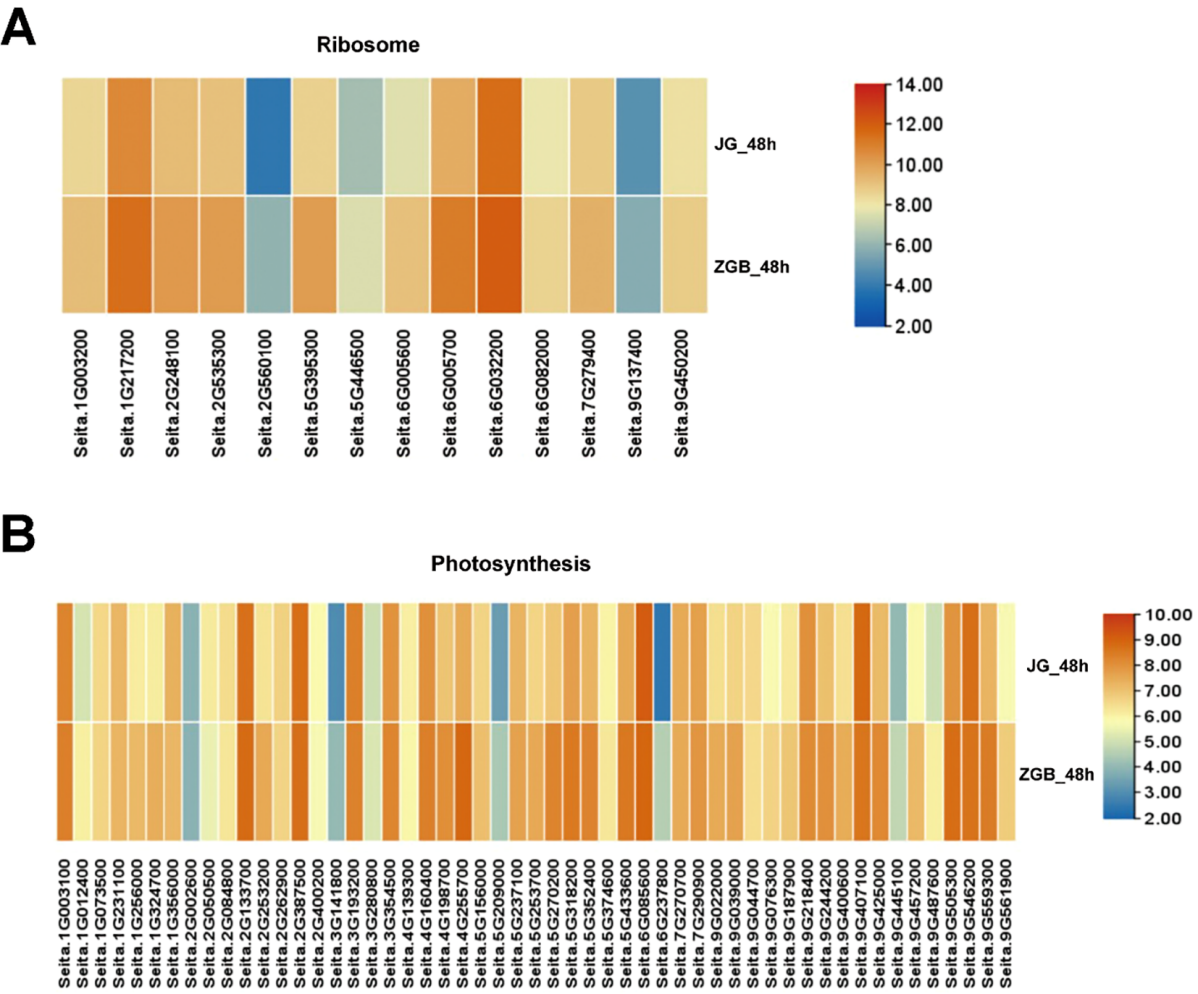
Analysis of the expression levels of genes involved in the Ribosome and Photosynthesis pathways. Participated in a heat map analysis of gene expression in the ribosome pathway at 48h in both varieties **(A)**. Participated in a heat map analysis of gene expression in the Photosynthesis pathway at 48h in both varieties **(B)**.

Genes associated with the ‘ribosome’ and ‘photosynthesis’ metabolic pathways were significantly down-regulated at 24h in ZGB and JG19. By 48h, they were significantly up-regulated in ZGB but not in JG19. We did a heat map analysis of the expression of genes related to photosynthesis and ribosomal pathways in ZGB and JG19, at 48h (**Figure 6**), and we can clearly see that the expression of related genes in ZGB varieties was higher than that in JG19. Therefore, we hypothesized that the expression of genes related to photosynthesis and ribosomes was positively correlated with the plant’s resistance to the disease, and that this was one of the reasons for the greater resistance of ZGB to the disease than that of JG19.

### 3.4 Analysis of disease resistance-related transcription factor family

**Figure 7:**
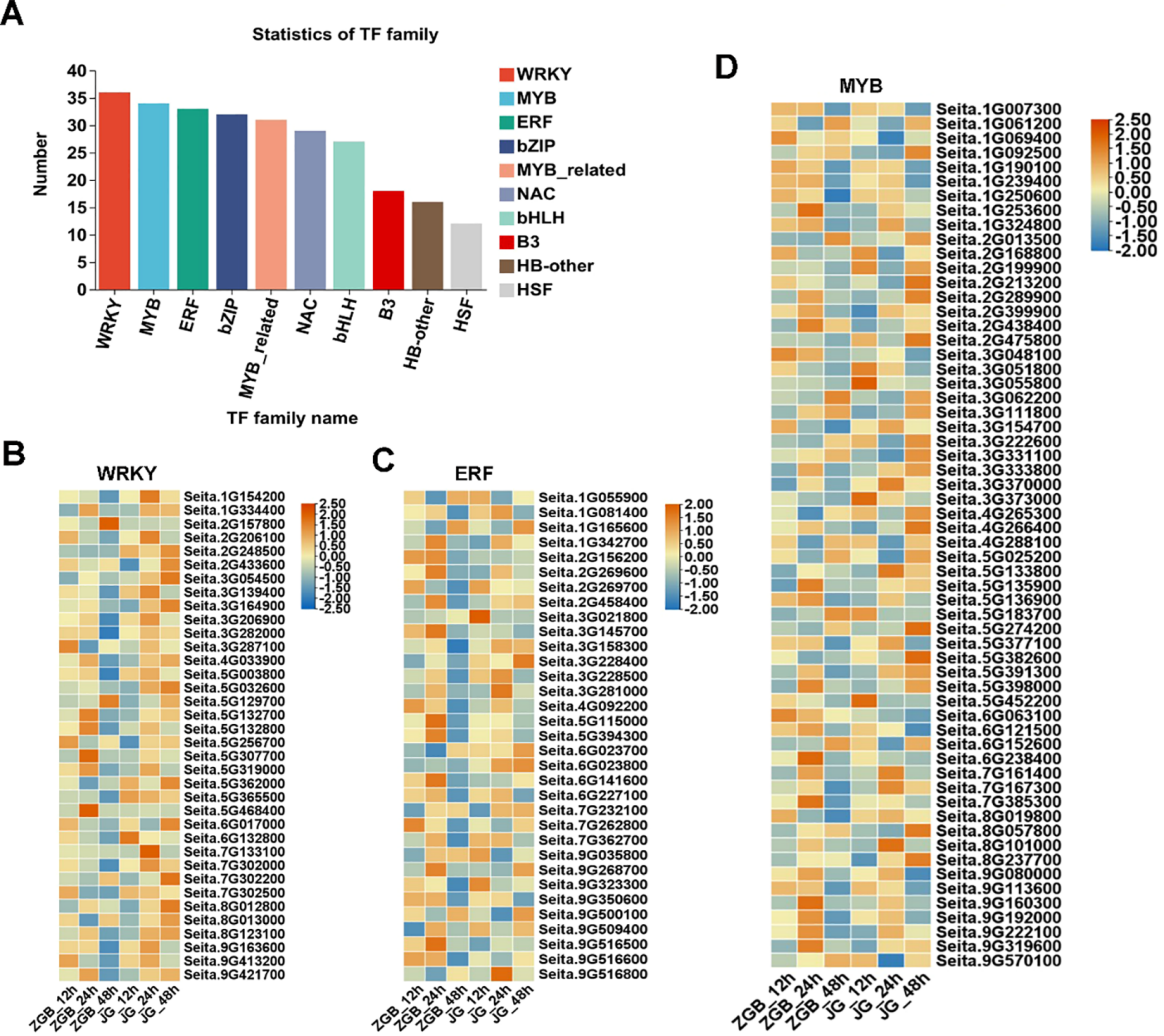
Analysis of transcription factor family expression levels in different varieties at different stages. Statistical analysis of transcription factors involved in ZGB 24h **(A)**. Heat map analysis of WRKY expression levels in different varieties at different stages **(B)**. Heat map analysis of ERF expression levels in different varieties at different stages **(C)**. Heat map analysis of MYB expression levels in different varieties at different stages **(D)**.

Transcription factor prediction of genes was performed using the transcription factor database Plant TFDB, and the obtained transcription factor families were counted according to the transcription factor prediction results, and it was found that the predicted transcription factors belonged to 40 families of TFs at ZGB 24 hip (**Figure 7A**), and the three families with the highest number of genes, namely, WRKY, MYB, and ERF, were further analyzed, and their expression in other periods and in the expression in JG19 were analyzed (**Figure 7B**). Among the genes up-regulated at 24h in ZGB, 36 genes were enriched in the WRKY family, 34 in the MYB family and 33 in the ERF family, and the expression of genes enriched in the MYB family was higher than that in the other two families. At 24h in both varieties, the expression levels were higher than the other periods. Over all the expression at 24h in ZGB was higher than at 24h in JG19.

### 3.5 Analysis of disease resistance-related MAPK pathway

**Figure 8:**
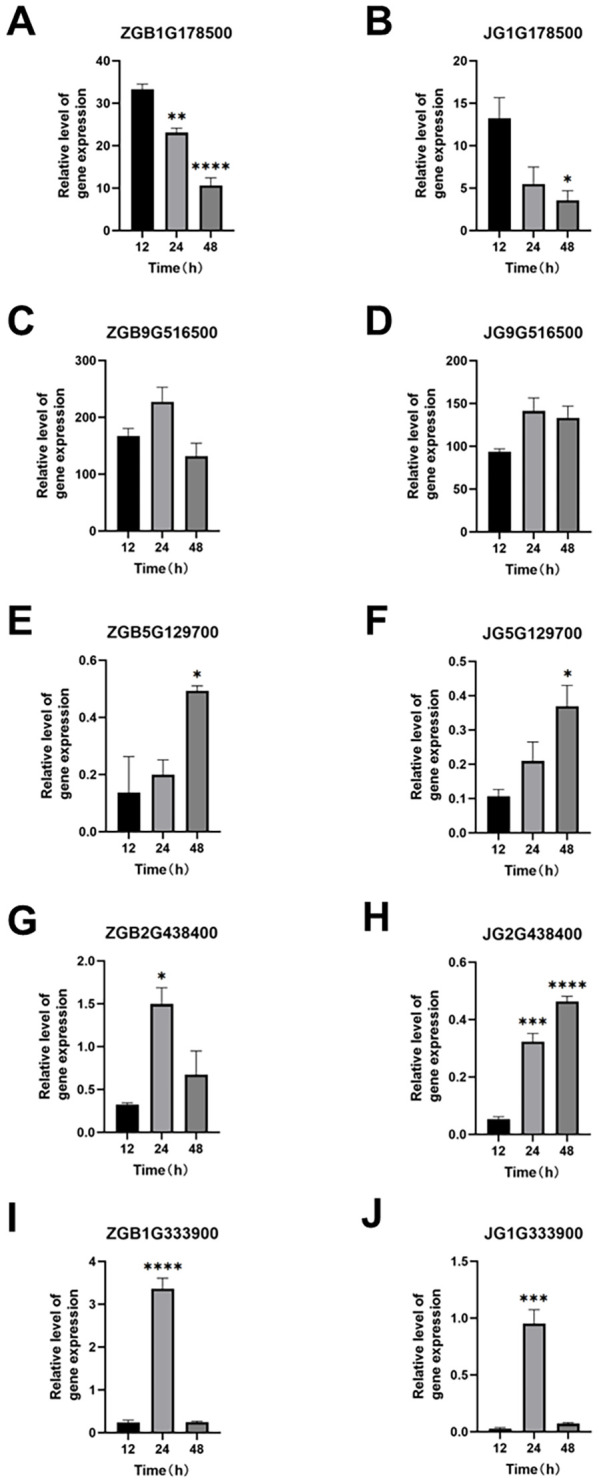
Real-time qPCR validation of representative genes at three time points in ZGB and JG19 during infection by *Ps3*. The left Y axis corresponds to the gene expression level in transcriptome sequencing shown in broken lines. The right Y axis corresponds to the relative gene expression level in qRT-PCR shown in a column. Reference gene: β-actin; n = 3 **(A–J)**. *p < 0.05, **p < 0.01, ***p < 0.001, ****p < 0.0001 (compared with the control group, using Student’s t-test).

**Figure 9:**
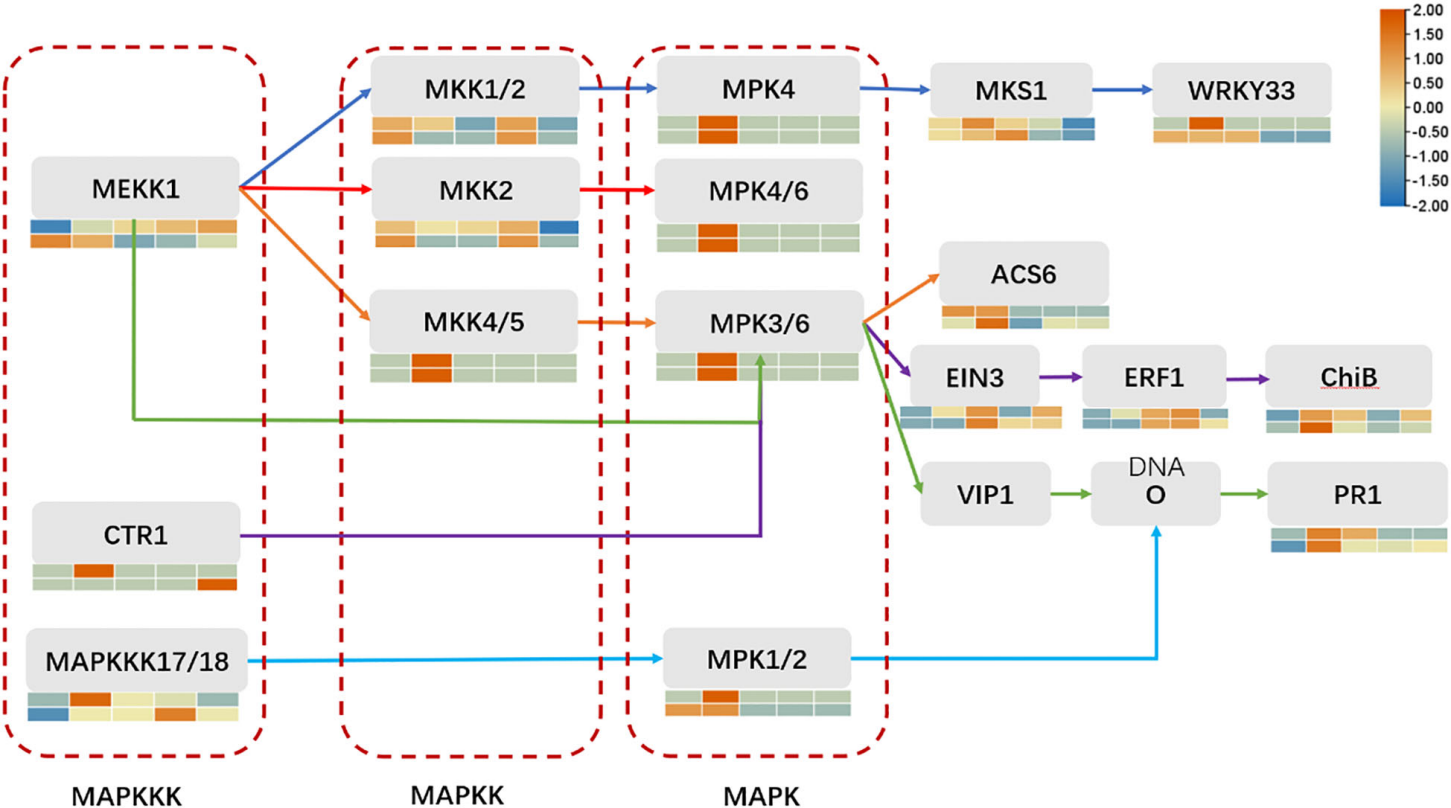
MAPK pathway map and expression level analysis of genes involved in ZGB expression at 24 hours in other periods and varieties. From top to bottom, they are ZGB and JG19. From left to right, they are 12h, 24h, 36h, 48h, and 72h.

The basic components of the MAPK pathway include MAPK kinase kinase (MKKK), MAPK kinase (MKK), and MAPK, which can be activated sequentially, and the MAPK pathway in plants is one of the common intersecting pathways of various signaling pathways such as cell division and differentiation, hormone response, and adversity response, and plays a pivotal role in the growth and development of plants. Through our analysis of transcription factors mentioned above, we hypothesized that the MAPK metabolic pathway affects the activity of some downstream related transcription factors, so we found the genes involved in the MAPK pathway during the period of ZGB 24 hip and analyzed the expression levels of these genes during other periods of time in different varieties (**Figure 8**). Firstly, in MAPKKK, mainly MEKK1, CTR1 and MAPKKK17/18, then to MAPKK, mainly involved in MKK1/2, MKK2 and MKK4/5, and secondly to MAPKK, mainly MPK4, MPK4/6, MPK3/6 and MPK1/2, and finally to activate some downstream related transcription factors and genes. Among them, the genes related to the regulation of MEKK1 were expressed throughout the whole period of ZGB and JG19, and the genes related to the regulation of MKK4/5, MPK4, MPK4/6, and MPK3/6 were expressed only in the middle period (24h) of ZGB and JG19. In addition, overall, most genes involved in the MAPK pathway were expressed at significantly higher levels in the middle of ZGB and JG19. And most of the ZGB had higher expression levels than JG19 in the same period, which we speculated might be related to the disease resistance of plants. We further focused on the core components of the MAPK signaling cascade (1G178500, 1G333900) as well as key immune-related transcription factors, including WRKY (5G129700), ERF (9G516500), and MYB (2G438400). By integrating transcriptomic and qRT-PCR data (**Figure 9**), we conclude that the MAPK signaling pathway is rapidly and robustly activated at an early stage in the resistant variety ZGB. Notably, the MAPK component genes (1G333900, 1G178500) were significantly upregulated at 24 hours post-inoculation (24 hip). Further analysis revealed that MEKK1-related genes exhibited sustained expression throughout the time course, likely contributing to basal defense regulation, whereas the MKK4/5–MPK3/6 module was specifically induced at 24 hip, implicating its role in ZGB-specific resistance signaling. Downstream transcription factors displayed coordinated activation: WRKY (5G129700) and ERF (9G516500) were markedly upregulated in ZGB, with expression kinetics aligning with the expected MAPK-transcription factor regulatory cascade. Meanwhile, the persistent high expression of MYB (2G438400) suggests its potential involvement in reinforcing structural defenses through modulation of secondary metabolic pathways. Critically, all validated genes showed significantly higher expression levels in ZGB compared to the susceptible variety JG19, consistent with RNA-Seq results (concordance rate: 90.91%), and their expression peaks clustered at 24 hip—precisely coinciding with the phenotypically observed critical window of resistance. These findings collectively provide molecular evidence for the central role of the MAPK signaling pathway and associated transcription factors in conferring resistance to foxtail millet blast.

## 4 Discussion

In current life science research, RNA-Seq technology continues to play a crucial role in various plant research studies. Especially, it is emerging as a key “weapon” for uncovering plant disease resistance genes, providing solid technical support for a deeper understanding of the complex and sophisticated molecular interaction mechanisms between plants and pathogens, and demonstrating unparalleled application potential. For instance, through transcriptome analysis, the resistance mechanism of rice to bacterial leaf blight (Rice bacterial leaf blight, BLB) has been successfully revealed. It was found that the expression levels of NLR protein-coding genes *LOC_Os07g02560* and *LOC_Os07g02570* in resistant rice varieties were significantly higher than those in susceptible varieties, providing key gene targets for the study of rice resistance to BLB (Shu et al., 2021). In the field of wheat research, researchers have used this technology to analyze the resistance mechanism of wheat to root rot fungus (*Bipolaris sorokiniana*), precisely identifying multiple defense-related genes such as *Cre3*, *EDS1*, *LTP5*, *PGIP*, *PR-1*, *PIEP1*, *TLP*, *UGT*, *Stb6*, and *PFT* (Qalavand et al., 2023). This has revealed the complex defense mechanism in which wheat utilizes a “cross-pathogen resistance gene pool” and reactive oxygen species scavenging systems to work together and defend against pathogens opening up a new path for studying wheat’s resistance to root rot. Using RNA-Seq high-throughput sequencing technology *Bs4C*-a gene for resistance to *Xanthomonas*-which regulates recognition of *Xanthomonas* effector proteins—was isolated from chili peppers. In researching disease resistance in millet transcriptome sequencing technology also plays an indispensable role. Through RNA-seq technology researchers have found that after *Sclerospora graminicola* infects millet it changes normal expression patterns of related genes in millet including MYB domain protein (*Si2g07390*), ethylene response transcription factor ERF094 (*Si8g10010*), and NAC domain protein (*Si5g40460*) thereby inducing abnormal transformation of inflorescence organs back into leaves causing abnormal development of millet flower organs and fruiting obstacles which seriously affects yield and quality in millet (Sun et al., 2024). However there is still a large gap in research related to millet blast. To fill this gap, we used transcriptome sequencing technology conduct an in-depth analysis on differences between resistant and susceptible varieties’ transcriptional levels It was found that genes responding to millet blast showed extremely active expression after infection with numbers involved gradually increasing as infection progressed. Especially during 24–36 hours post infection; significant differences of gene expression were observed. It indicates that 24–36 hours after the pathogen infection is the crucial stage for the development of resistance. This result provides key clues for further investigating self-repair abilities among resistant versus susceptible varieties.

**Figure 10:**
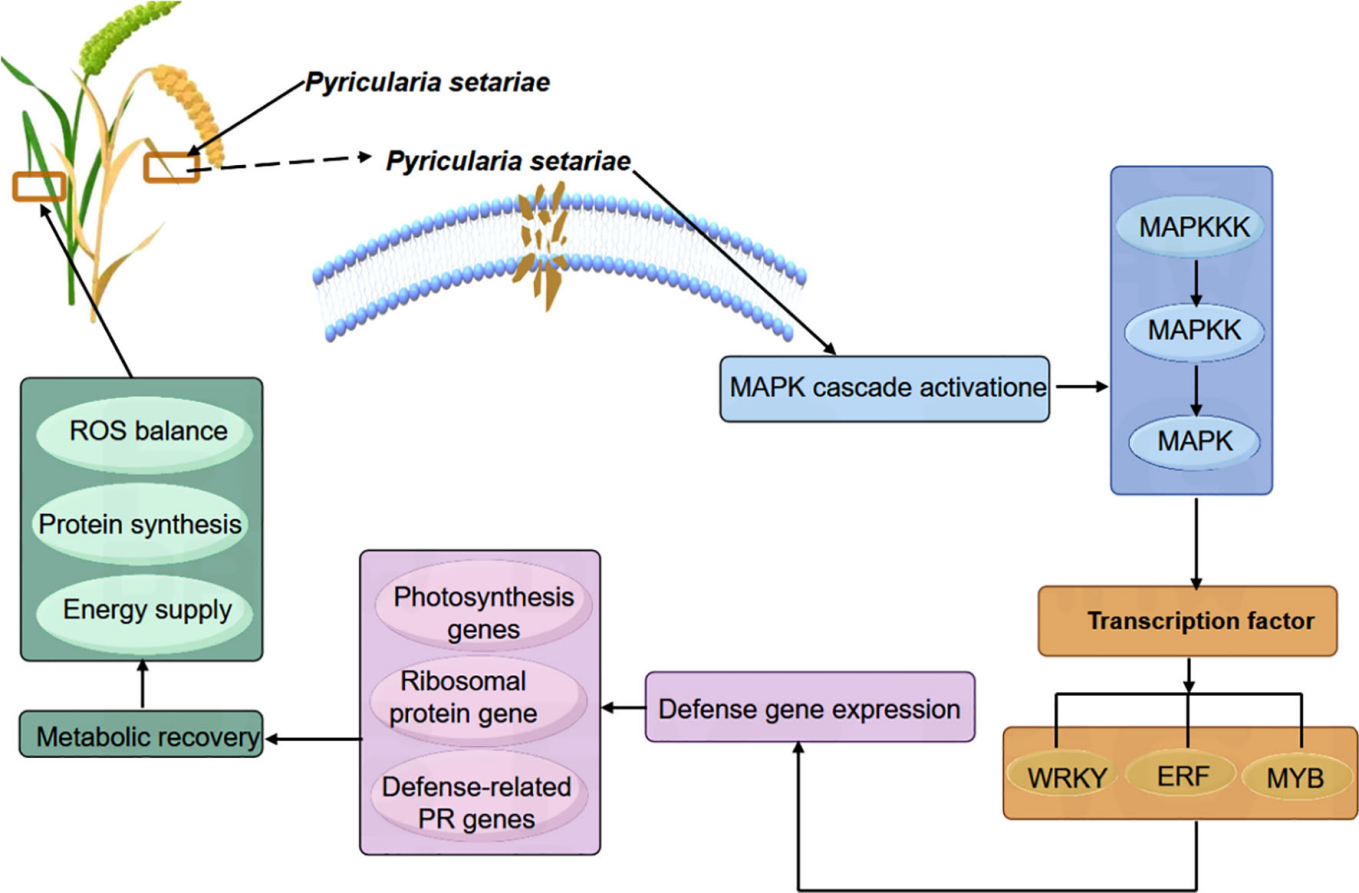
Pattern diagram of MAPK pathway regulating the response of millet to *Ps3*.

A substantial body of evidence has established the MAPK (mitogen-activated protein kinase) signaling pathway as a central component of plant immunity, functioning through a conserved three-tiered cascade—MAPKKK–MAPKK–MAPK—to mediate disease resistance across diverse plant species. This study reveals that although the resistant foxtail millet variety ZGB and the susceptible variety JG19 exhibit similar overall expression patterns of MAPK-related genes during *Ps3* infection, indicative of the pathway’s fundamental conservation in immune responses, they differ significantly in the timing of upstream signal activation and systemic defense regulation. During the early infection phase (0–12h), both varieties show widespread downregulation of MAPK pathway components and multiple metabolism-associated genes, likely reflecting an initial stress response triggered by pathogen colonization. However, at the critical stage of infection (24–36h), ZGB rapidly initiates a robust immune response: key MAPKKK genes such as MEKK1 and CTR1, along with transcription factors including WRKY and ERF, are significantly upregulated; the MAPK cascade (MKK4/5, MPK3/6) is strongly activated; and downstream transcriptional networks are subsequently engaged. Concurrently, photosynthesis- and ribosome-related metabolic pathways are strategically suppressed, indicating a deliberate reallocation of cellular resources toward immune defense. In contrast, JG19 fails to mount effective early signal activation or metabolic reprogramming. In the recovery phase (48–72h), ZGB exhibits pronounced self-repair capacity, with the reactivation of photosynthetic and ribosomal protein-related genes restoring ROS homeostasis, protein synthesis, and energy production, while sustained expression of MAPK-dependent defense genes maintains a prolonged state of resistance. This temporally coordinated immune regulatory program aligns closely with the conserved roles of the MAPK pathway in other crops: for example, *GhMYB36* activates PR1 to coordinate stress responses (Liu et al., 2022); *CsWRKY65* constructs a ROS-PR cooperative network to enhance fruit disease resistance (Wang et al., 2021) and *OsBIERF3* modulates PR and MAPK genes to confer broad-spectrum resistance (Hong et al., 2022). This study systematically demonstrates that ZGB’s enhanced resistance stems from early activation of the MAPK cascade, leading to phosphorylation of downstream transcription factors—including WRKY, ERF, and MYB—which in turn regulate the expression of photosynthesis- and ribosome-related genes, facilitating tissue repair and immune homeostasis restoration (**Figure 10**). This mechanism mirrors the well-characterized Arabidopsis model in which ROS accumulation activates the MAPKKK *AtANP1*, initiating a phosphorylation cascade that establishes systemic resistance. Collectively, the superior disease resistance of ZGB over JG19 is attributed to its precisely orchestrated temporal control of signal initiation, resource allocation, and metabolic remodeling.

As autotrophs, plants convert light energy into chemical energy through photosynthesis to synthesize the sugars they need. However, heterotrophic plant pathogens must rely on the sugar resources of their host plants to complete their life cycles. This has led to intense competition between plants and pathogens for the limited sugar resources. Plants defend against infection by restricting the pathogens’ access to sugars, while pathogens must compete for these resources to survive and reproduce (Chen et al., 2024). To disrupt the host’s photosynthesis, some pathogens directly target chloroplasts, altering the structure of thylakoid membranes and inhibiting the production of key defense signaling molecules such as reactive oxygen species (ROS), nitric oxide (NO), and salicylic acid (SA). Successful pathogen infection disrupts the host’s physiological and metabolic functions, with photosynthesis often being one of the first processes affected. Ribosomes, as core machinery for protein synthesis, are responsible for synthesizing a large number of disease-resistant proteins in plant defense responses. In recent years, chloroplast immunity has become a hot topic in studying disease resistance mechanisms and plays an important role in plant defense. Studies have shown that both photosynthesis and ribosome metabolism are closely related to plant disease resistance. Disease-resistant plants can maintain homeostasis of reactive oxygen species (ROS) by regulating the expression of photosynthesis-related genes, thereby enhancing their disease resistance (Qi et al., 2024). For example, enhancing the expression of potato chloroplast extension factor *StTuA/B* can enhance potato photosynthesis, increase yield, and also enhance potato’s resistance to *Phytophthora infestans* (Qi et al., 2024). rice transcription factor *OsDREB1C* has been found to be a multifunctional regulatory factor that determines photosynthetic capacity; its overexpression can significantly increase crop yield, nitrogen use efficiency, and shorten growth period (Wei et al., 2022). The wheat ribosome-related gene *TaRsfS* has been discovered to negatively regulate powdery mildew and stripe rust resistance by inhibiting activity of key enzyme *TaOPR1* in jasmonic acid (JA) synthesis; its gene editing can significantly improve wheat disease resistance (Li et al., 2025). In an in-depth analysis of transcriptome data, we found that 36 hours after pathogen infection there were significant differences in self-repair capabilities between resistant and susceptible varieties. Specifically, ZGB variety could actively mobilize expression of photosynthesis- related and ribosome-related genes to resist pathogen attack while JG19 variety showed continuous down-regulation of these genes. This indicates that ZGB can more effectively activate its defense mechanisms in response to pathogen attack and achieve self-repair within 36 hours while self-repair ability of JG19 variety is relatively weak.

## 5 Conclusions

This study reveals that the superior resistance of foxtail millet to *Pyricularia setariae* is attributed to the rapid and robust activation of the MAPK signaling pathway during the critical 24–36 hours post-inoculation. In the resistant variety ZGB, this activation coincides with a transient downregulation of photosynthesis- and ribosome-related genes, followed by a swift recovery—highlighting a dynamic strategy of resource reallocation and metabolic self-repair. In contrast, the susceptible variety JG19 exhibits a similar initial suppression but fails to restore these essential pathways in the later stages. Furthermore, the amplified MAPK cascade in ZGB drives the upregulation of key downstream defense-related transcription factors—including WRKY, ERF, and MYB—orchestrating a coordinated and effective immune response. Our findings establish the MAPK pathway as a central regulator of early plant immunity. This study provides theoretical and technical support for the early diagnosis, prevention and control of *P. setariae* and the breeding of resistant *S. italica* varieties.

## Data Availability

The data presented in the study are deposited in the NCBI BioProject, accession number PRJNA1201740.
