# Supplementary material for: Transcriptome analysis reveals early activation of MAPK pathways involved in the resistance of Setaria italica against Pyricularia setariae
Source: Front Plant Sci. 2025 Nov 27;16:1676191. doi: 10.3389/fpls.2025.1676191 (PMC12695818; doi:10.3389/fpls.2025.1676191)
Supplement: Supplementary file 3 [file Table1.docx]

**Table S1 Primers Used in This Study.**

| Primer | Sequence (5′ to 3′ ) | Usage |
| --- | --- | --- |
| SiActin_F/R | CGCATATGTGGCTCTTGACT/GGGCACCTAAATCTCTCTGC | RT-qPCR |
| JG2G230500-F/R | CAAGAAGCTCACCTCCGCCA/CTCGTGGGAGACCAACCTGC |  |
| JG5G361200-F/R | GCGCAGAGCTTCGAGTACAA/GACGTGTCTCCTTGACCTCC |  |
| JG6G178500-F/R | GGGGCTACACCATGAAAGGA/ACTTGTTGGGCTTCTCTGGG |  |
| JG3G179700-F/R | CAACGCTGCCGGTATAAACG/TCGCTTTCTTTCTCGCACCT |  |
| ZGB6G08680F/R | GGAAGGGCTCCTCAACAACA/AGCTCGAAGATGATGGGCTG |  |
| ZGB9G20630F/R | CCTTTCTGGCCTCTGCTTCC/CTGTACCCCTGCCCCATGAC |  |
| ZGB5G14070-F/R | CCCAAGGGACGTGGAGTTCA/AAGTCGAGCCCGTCACAGC |  |
| ZGB2G05900-F/R | GCAAGCGGGCATTTACCATC/CCTGTTCGTTCAGCAACCTG |  |
| 1G333900-F/R | GGAGATCGTGCTTCTGAGCC/GAGGCAAATGTGCTGAGGTG |  |
| 1G178500.2-F/R | GCAGCTGTTGGTTTCAAGGG/GCAGCTGTTGGTTTCAAGGG |  |
| 9G516500-F/R | TCCCGGATCGAGGAAGAACA/TTGATCAACTCAAGCAGTCAGT |  |
| 5G129700-F/R | AAGGTCGTCAAGAACACGCA/TTGGGAGCAAATGTGCAAGT |  |
| 2G438400-F/R | GTTTCTGCTGCTTGCTTTCCT/CATGTGTGCAGTGCAGGTGG |  |
